# The chloroplast and mitochondrial genomes of the green algae *Pediastrum duplex* isolated from Central Georgia (USA)

**DOI:** 10.1080/23802359.2019.1666666

**Published:** 2019-09-19

**Authors:** Grayson C. R. Proulex, Blia Lor, Kalina M. Manoylov, A. Bruce Cahoon

**Affiliations:** a Department of Natural Sciences, The University of Virginia’s College at Wise, Wise, VA, USA;; b Department of Biological and Environmental Sciences, Georgia College and State University, Milledgeville, GA, USA

**Keywords:** Green algae, Hydrodictyaceae, *Pediastrum*, chondriome, plastome

## Abstract

A *Pediastrum duplex* (Chlorophyta) strain was isolated from a freshwater system in Milledgeville, Georgia and its chloroplast and mitochondrial genomes sequenced. The chloroplast genome was 199,241 bp with 136 genes and the mitochondrial 40,756 bp with 40 genes, both were circular. Comparison of the ‘Milledgeville’ plastome to other *P. duplex* isolates revealed a nearly identical sequence identity to archived genes and genomic fragments from the strain UTEX1364 which was isolated from Lake Machovo in 1962. These sequences provide chloroplast and mitochondrial genomes from a wild *P. duplex* isolate and provide more organelle genomes for a genus with cryptic phylogenetic relationships.

*Pediastrum duplex* Meyen (Chlorophyta) is a common freshwater green microalga that grows as circular disc-like colonies with outer cells having two prominent lobes (Guiry and Guiry 2019). This genus contains colonial green algae with a distinctive morphology but ambiguous phylogenetic relationships (McManus and Lewis 2011; Fučíková et al. 2014). Attempts to resolve these ambiguities have led to the sequencing of complete mitochondrial (Farwagi et al. 2015) and chloroplast (McManus et al. 2018) genomes of representative species. Despite the increase in genetic data, the genus Pediastrum remains difficult to resolve. In this study, we present the organellar genomes from a newly isolated *P. duplex* strain.

A *Pediastrum* was collected on February 25, 2015 from a water pool in a greenway park that runs adjacent to the Oconee river in Milledgeville, GA (Lat: 33.10°N, Long: 83.20°W), cultured to near purity (some bacteria still present) in Bold Modified Basal Freshwater Nutrient Solution (B5282-500ML, Sigma-Aldrich, St. Louis, MO), and identified as *P. duplex*. The type material was deposited in the Georgia College and State University Natural History Museum, USA diatom collection, GCGC1003.

The total DNA was isolated using Qiagen’s DNeasy kit for plants (Valencia, CA). A 150 bp library was prepared and sequenced using Illumina MiSeq technology by Genewiz (South Plainfield, NJ). The complete chloroplast and mitochondrial genomes were assembled using the *de novo* assembly algorithm embedded in Geneious Prime v. 2019.0.3 (BioMatters, Ltd., Auckland, New Zealand). A single full-length candidate sequence was produced for the plastome. Two candidate sequences were assembled for the chondriome but PCR assays only confirmed one of them. Sequences have been deposited into GenBank: plastome – MK895950, chondriome – MK895949. Raw sequence data were deposited into the National Center for Biotechnology Information (USA) Sequence Read Archive – accession PRJNA545453 (Figure 1).

**Figure 1.**
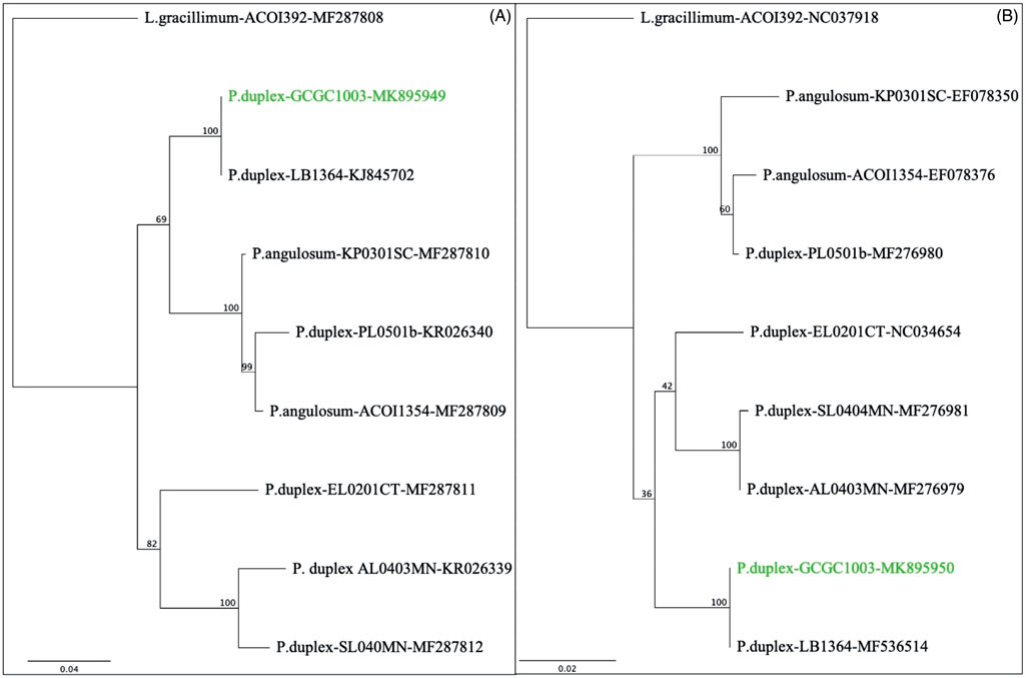
Phylogenies of the mitochondrial *coxI* gene (A), and the chloroplast *rbcL* gene (B), including *P. duplex* Milledgeville (green). Alignments were constructed using the Geneious Prime aligner. Phylogenies were constructed using PhyML in Geneious Prime, with the Jukes–Cantor substitution model and 100 bootstrap replicates. The first code to the right of the species name is the strain designation. The second code is the GenBank accession number. P.: Pediastrum; L.: Lacunastrum.

The Milledgeville, GA, isolates of *P. duplex* had a circular chloroplast genome, 199,241 bp with the common chloroplast genome structure of two inverted repeat (IR) regions separated by two single copy (SC) regions. It contained 69 protein-coding genes, 26 tRNA’s and 3 rRNA’s. The mitochondrial genome was circular, 40,756 bp, and contained 13 protein-coding genes, 24 tRNAs and 2 rRNA’s. The gene content of the organellar genomes was identical to other *Pediastrum* strains archived in GenBank. The chloroplast and mitochondrial coding regions of the ‘Milledgeville’ isolate had nearly 100% sequence identity with genes and genomic fragments from the strain LB1364 (UTEX algal culture collection) which was collected from Lake Machovo in 1962 in the modern Czech Republic.

The primary differences between the ‘Milledgeville’ isolate and other Pediastrum organellar genomes were in genome size and gene synteny. The expansions/contractions of intergenic regions appear to be common between different clades of Streptophyte green algae (Lemieux et al. 2016) but within a species this is rare. Both the chloroplast and the mitochondrial genomes of *P. duplex* provide evidence of intra-specific rearrangements. Other examples of similar rearrangements have been reported in green algal species, including *P. duplex* (Farwagi et al. 2015; Turmel et al. 2017; Kim et al. 2019).
